# Association between Tooth Extraction 2 Weeks before Radiotherapy and Osteoradionecrosis in Patients with Advanced Oral and Oropharyngeal Cancer

**DOI:** 10.7150/jca.97294

**Published:** 2025-07-28

**Authors:** Masatoshi Usubuchi, Seiki Kanemura, Atsushi Hozawa, Ichiro Tsuji

**Affiliations:** 1 Department of Dentistry, Miyagi Cancer Center, Japan.; 2 Division of Cancer Epidemiology and Prevention, Miyagi Cancer Center Research Institute, Japan.; 3 Division of Epidemiology, School of Public Health, Tohoku University Graduate School of Medicine, Japan.

**Keywords:** osteoradionecrosis, radiotherapy, tooth extraction, oral cancer, oropharyngeal cancer, risk factor

## Abstract

**Objective:** Osteoradionecrosis (ORN) is the most serious late adverse event in standard treatment for locally advanced oral and oropharyngeal cancers. The clinical guidelines recommend dental extractions be completed at least 2 weeks before the start of radiotherapy to reduce the risk of ORN development. However, to what extent tooth extraction 2 weeks before radiotherapy, as recommended by the guidelines, reduces the risk of ORN development is unclear. This study was conducted to examine the association between tooth extraction 2 weeks before radiotherapy and ORN development.

**Methods:** The study included patients aged ≥18 years who received chemoradiotherapy with 3-weekly cisplatin or radiotherapy alone for locally advanced oral and oropharyngeal cancer at the head and neck surgery of Miyagi Cancer Center in Japan between 2011 and 2018. Additional oral care (AOC) was provided to the patients between 2011 and 2014; however, because of the downsizing of dentistry in 2015, usual oral care (UOC) was provided to the patients between 2015 and 2018. In the AOC group, all dental infection foci and teeth with poor prognosis in the radiation field were removed 2 weeks before radiotherapy. The cumulative incidence of ORNs was calculated using the Kaplan-Meier method, and multivariate analyses were performed using the Fine-Gray model with death as a competing risk for ORN development.

**Results:** Ninety-three patients were analyzed, 43 in the AOC and 50 in the UOC. The cumulative incidence rate of ORN in the AOC group was lower than that in the UOC group (0.071 vs. 0.415, p < 0.001). The hazard ratio (HR) for the incidence of ORN in the AOC group versus that in the UOC group was lower (HR, 0.108, 95% CI 0.019-0.606). In the subgroup analysis, HRs were lower in the following groups: male (0.062, 0.009-0.425), Eastern Cooperative Oncology Group performance status 0 (0.141, 0.028-0.700), without diabetes (0.135, 0.029-0.635), drinkers with a Sake index of ≥60 (0.033, 0.002-0.518), advanced cancer of clinical stage Ⅳ (0.151, 0.025-0.909), concurrent chemotherapy (0.126, 0.022-0.702), total cisplatin dose of ≥200 mg/m^2^ (0.055, 0.007-0.411), and dental risk factors (0.061, 0.007-0.537).

**Conclusions:** This study showed that extraction of at-risk teeth 2 weeks before radiotherapy reduced the risk of ORN development by approximately 90%. However, these results are based on a retrospective observational study conducted at only one center. Thus, future multivariate studies conducted at multiple centers, with death as a competing risk, are needed.

## Introduction

The incidence of head and neck cancer is the seventh highest in the world, and oral and oropharyngeal cancers account for approximately 40% of them 1. The standard treatment for locally advanced oral and oropharyngeal cancer in unresectable cases or high risk postoperative recurrence is chemoradiotherapy with 3-weekly cisplatin (3w-CDDP + RT) or radiotherapy alone 2,3. Osteoradionecrosis (ORN) is the most serious late adverse event associated with these treatments, which is defined as bone exposure for 3 months without residual tumor or recurrence 4,5. ORN is more common within 3-4 years after radiotherapy and greatly reduces quality of life 6,7. The incidence of ORN has been reported to range from 0.92% to 40%, and the incidence is higher in oral and oropharyngeal cancers near the mandible than in other sites 7-11.

The following risk factors have been reported to increase the incidence of ORN: poor oral health 6,8,10-14, advanced age 8,15, diabetes mellitus 16,17, smoking 12,16,18,19, alcohol consumption 16,20, tumor location (oral) 6,7,11,16,19,21,22, advanced cancer 8,19, high total radiation dose 21-23, radiation modality (three-dimensional conformal radiation therapy: 3D-CRT) 18,19,23,24, concurrent chemotherapy 16,22,25, and surgery before radiotherapy^7^,^17^,^26^. Previous studies have classified risk factors into patient, cancer, and treatment factors 11,27.

It has been shown in various cohort studies that tooth extraction before radiotherapy prevents the ORN development^25^,^28^-^30^. Based on these findings, without randomized controlled trials (RCTs), the National Comprehensive Cancer Network (NCCN) published guidelines recommended dental extractions be completed at least 2 weeks before the start of radiotherapy to reduce the risk of ORN development in 2014^31^. After the publishment of the guidelines, it has become ethically difficult to conduct RCTs and only the results of observational studies have been reported, but the results have been inconsistent. Wang et al. reported a hazard ratio (HR) of 1.069 (95% confidence interval [CI] 0.968-1.007) for tooth extraction before radiation for ORN 6, Liao et al. reported an HR of 1.168 (1.021-1.336)^26^, and Beech et al. reported an odds ratio of 5.19 (1.15-23.42) 32. On the other hand, Kojima et al. reported an HR of 0.62 (0.27-1.47) 21. In 2022, Normando et al. reported the effectiveness of tooth extraction before radiotherapy in a meta-analysis^33^. In 2023, the NCCN published guidelines and continued to recommend tooth extraction 2 weeks before radiotherapy 34. In Japan, however, there continue to be no recommendations regarding tooth extraction before radiotherapy 35,36. To what extent tooth extraction 2 weeks before radiotherapy, as recommended by the guidelines, reduces the risk of ORN development is unclear. The NCCN guidelines recommend the timing of tooth extraction, but do not specify clear criteria for tooth extraction. Therefore, this study aimed to evaluate the extent to which our institutional protocol for tooth extraction, the criteria for which are detailed in the Methods section, could reduce the risk of ORN.

In addition, ORN is not observed when death occurs without ORN, death can be considered a competing risk for ORN development. Liao et al. reported the association between tooth extraction before and after radiotherapy and ORN development 26, calculating death as a competing risk; however, no other study has analyzed death as a competing risk. Several reports have analyzed smoking and alcohol consumption as covariates, which are risk factors for ORN development 11,17,19. However, to our knowledge, no studies have analyzed death as a competing risk and smoking and alcohol consumption as continuous variables in multivariate analysis.

The Miyagi Cancer Center (MCC) in Japan has provided routine oral care to patients with oral and oropharyngeal cancer before receiving radiotherapy, and since 2011, based on the dental risk factors (DRFs) and malignancy-related risk factors (MRRFs) described previously by Schiødt M et al. 37, we have added the removal of dental infections and poor prognosis teeth contained in the high-dose radiation field 2 weeks before radiotherapy. However, since 2015, due to the downsizing of dentistry, no tooth extractions have been performed, and only routine oral care has been provided. Thus, we can clearly distinguish the periods in which the teeth were extracted before radiotherapy and those in which they were not.

We used this unique data to examine the association between tooth extraction 2 weeks before radiotherapy and ORN development. In the analysis, death was analyzed as a competing risk for ORN development, and risk factors for ORN development, such as smoking and alcohol consumption, were used as covariates.

## Materials and Methods

This study was approved by the Ethical Review Committee of MCC (Approval no. 2018-11) and was conducted in compliance with the Declaration of Helsinki. The information of the study was disclosed on our website and participants were given the chance of opt-out from the study at their own will.

We conducted a retrospective observational study reviewing electronic medical records. Patients with locally advanced oral and oropharyngeal cancer aged ≥18 years who received radiotherapy alone or 3w-CDDP + RT at the head and neck surgery of MCC between September 1, 2011, and March 31, 2018, were included in the study. As noted above, additional oral care (AOC) was provided to the patients between September 2011 and December 2014 (AOC group) and usual oral care (UOC) to the patients between January 2015 and March 2018.

### Oral care methods

Before radiotherapy, all patients were referred to the dentistry department of MCC for a basic periodontal examination, panoramic X-ray or computed tomography, oral hygiene instruction, and professional dental cleaning with scaling and tooth polishing. In the AOC group, all dental infected foci and teeth with poor prognosis in the radiation field were removed 2 weeks before radiotherapy. This was a modification of the method previously described by Schiødt M et al. 37, in which removal was performed when both MRRFs and DRFs were met. Infected foci were defined as dental infection foci. MRRFs included the total radiation dose of 40 Gy, molar teeth within the irradiation field, or teeth close to the tumor (1.0-1.5 cm). The following definitions of DRF described by Schiødt M. et al. 37 were used: severe caries that could not be treated conservatively (C4: 4th degree caries, caries reaching the dental pulp), root caries of more than 1/2 of the roots, untreated pulpitis or periapical caries, periodontal pocket ≥6 mm, cystic lesions, or filled teeth that were not completely bone filled. In the AOC group, all infected foci and teeth with poor prognosis in the radiation field were removed 2 weeks before radiotherapy. During radiotherapy, all patients brushed their teeth as regularly as possible every day and received professional teeth cleaning and oral mucosa rinsing once a week at the dentistry department of MCC according to the same protocol. After radiotherapy, all patients received regular follow-ups for cancer at the head and neck surgery department of MCC as well as regular follow-ups for oral care at the dentistry department or a family dentist according to the same protocol. The UOC group had the same dentist and frequency of practice as the AOC group. Follow-up oral care was similar for both dentists: every 1-2 months in the first year, every 3-4 months in the second year, and every 4-6 months in the third and fifth years.

### Methods of chemoradiotherapy and radiotherapy

According to the same protocol, radiation was administered at 2 Gy per dose, once a day, 5 days a week, for a total of 20-23 doses of 40-46 Gy to level I-IV lymph nodes for oral cancer and to level II-IV lymph nodes for oropharyngeal cancer. In cases of high risk for postoperative recurrence, additional irradiation was administered to extranodal infiltrating lymph nodes and areas of positive surgical margins with a margin of 1-1.5 cm. In unresectable cases, additional irradiation was administered to metastatic lymph nodes and the primary tumor within a margin of 1-1.5 cm. The total number of irradiation was 30-35 times, with a total dose of 60-70 Gy.

From September 2011 to October 2013, radiotherapy with 3D-CRT was performed. After November 2013, when intensity-modulated radiation therapy (IMRT) was initiated, IMRT and 3D-CRT were performed concurrently.

In the case of 3w-CDDP + RT, CDDP 100 mg/m^2^/body was administered intravenously on days 1, 22, and 43 of radiotherapy according to the same protocol.

### Primary endpoint

The primary endpoint was ORN development, and patients were observed from the date of radiotherapy completion to day 1,500. When a family dentist suspected ORN during the regular follow-up period after radiotherapy, the patient was referred to the dentistry department of MCC for a diagnosis of ORN. ORN was determined by doctors of the head and neck surgery department at MCC and a single dentist (Usubuchi) according to the following definition by Epstein 4: bone exposure in the radiotherapy area for 3 months or 90 consecutive days, with no evidence of residual or relapse tumor.

### Excluded patients from the analysis

**Figure 1:**
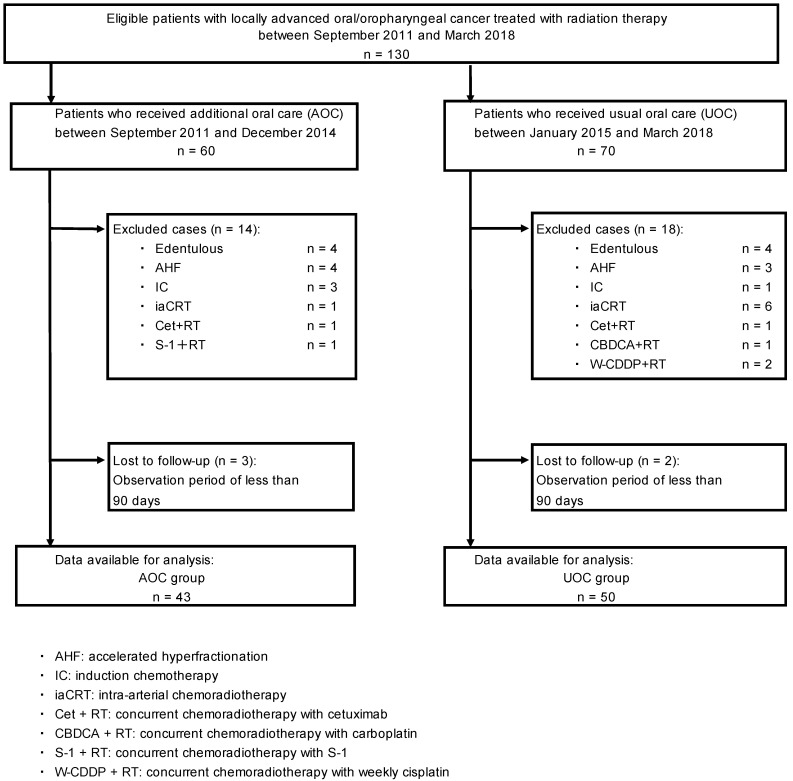
Patient flow in the study.

Dentulous patients and those who received treatment other than radiotherapy alone or 3w-CDDP + RT were excluded from the analysis (Figure 1). Patients with an observation period of <90 days were excluded because they could not be observed for 90 days, which meets the definition of ORN.

### Covariates

The following information considered factors associated with ORN development was collected from the electronic medical records: sex, age, Eastern Cooperative Oncology Group performance status (ECOG-PS)^36^, diabetes mellitus, smoking (Brinkman index), alcohol drinking (Sake index: multiplying the average daily alcohol intake in terms of 23 g of ethanol by the duration of drinking (years)) 37, location of the primary tumor, clinical stage (UICC TNM Classification of Malignant Tumors 7th edition) 38, radiation modality (3D-CRT/IMRT), total radiation dose (Gy), concurrent chemotherapy (3w-CDDP + RT), total cisplatin dose (mg/m^2^), surgery before radiotherapy, DRF, and tooth extraction after radiotherapy.

### Statistical analysis

Comparisons of patient characteristics and observations between the UOC and AOC groups were performed using Fisher's exact test for categorical variables and Wilcoxon's rank sum test for continuous variables. The cumulative incidence of ORNs was calculated using the Kaplan-Meier method, and comparisons between the two groups were performed using the log-rank test and generalized Wilcoxon test. Overall survival was also compared between the two groups using the Kaplan-Meier method with the log-rank and generalized Wilcoxon test. The cumulative incidence of ORNs with death as a competing risk of ORN development was also compared between the two groups using Gray's test.

Multivariate analyses were performed using the Fine-Gray model with death as a competing risk for ORN development, and HRs and 95% CIs for ORN development between the AOC and UOC groups. Three models were used. Model 1 was adjusted for patient factors such as sex, age, ECOG-PS, diabetes mellitus, Brinkman index, Sake index, and DRF. Model 2 was adjusted for cancer factors such as the location of the primary tumor and clinical stage in addition to model 1. Model 3 was adjusted for treatment factors such as radiation modality and concurrent chemotherapy in addition to those in model 2.

To evaluate the predictive ability of mean mandibular dose for ORN development, a logistic regression analysis was first performed with ORN development as the dependent variable and mean mandibular dose as the independent variable. Subsequently, Receiver Operating Characteristic (ROC) curve analysis was conducted based on the predicted probabilities from this logistic regression model. The area under the ROC curve (AUC) was calculated for all patients and separately for the UOC and AOC groups.

Subgroup analyses were performed to examine HRs for ORN development by various factors. The HRs and p-values for interactions were calculated using the multivariate analysis of model 3. HRs were not calculated if ORN did not developed in the subgroup.

SAS version 9.4 (SAS Institute, Inc., Cary, North Carolina, USA) was used for statistical analysis. Calculation of the cumulative incidence of ORNs and Gray's test were performed using the eventcode option of the LIFETEST procedure. The Fine-Gray model was performed using the eventcode option of the PHREG procedure. Two-tailed tests were used for all tests, and p < 0.05 was considered statistically significant.

## Results

Table 1 shows the basic characteristics and the results of the observations of the analyzed patients. Of the 93 patients, 72 (77.4%) were male, the median age was 64 years, 85 (91.4%) had ECOG-PS 0, 14 (15.0%) had diabetes mellitus, the median Brinkman index was 600, the median Sake index was 60, and no differences were found between the two groups. The primary tumor was located in the oral cavity in 40 (43.0%) patients, with a higher incidence in the AOC group than in the UOC group (55.8% vs. 32.0%, p = 0.023). Moreover, 73 (78.5%) patients had stage IV cancer, with no difference between the two groups. In addition, 53 patients (57.0%) received radiotherapy with IMRT, with higher frequency in the UOC group than in the AOC group (80.0% vs. 30.2%, p < 0.001). The median total radiation dose was 70.0 Gy. In total, 67 (72.0%) patients received concurrent chemotherapy, and the median total cisplatin dose was 200 mg/m^2^. In total, 51 (54.8%) patients underwent surgery before radiotherapy, and 73 (78.5%) had DRFs. No difference in the total radiation dose, concurrent chemotherapy, total cisplatin dose, surgery before radiotherapy, or DRF was found between the two groups. No tooth extraction was performed after radiotherapy in either group. The median observation period was 1,500 days for both groups. Moreover, 23 deaths were recorded during the observation period, with 17 (39.5%) patients in the AOC group and 6 (12.0%) in the UOC group, more in the AOC group (p = 0.003). ORN developed in 23 patients, with 3 (7.0%) in the AOC group and 20 (40.0%) in the UOC group, and the incidence was lower in the AOC group (p < 0.001).

**Figure 2:**
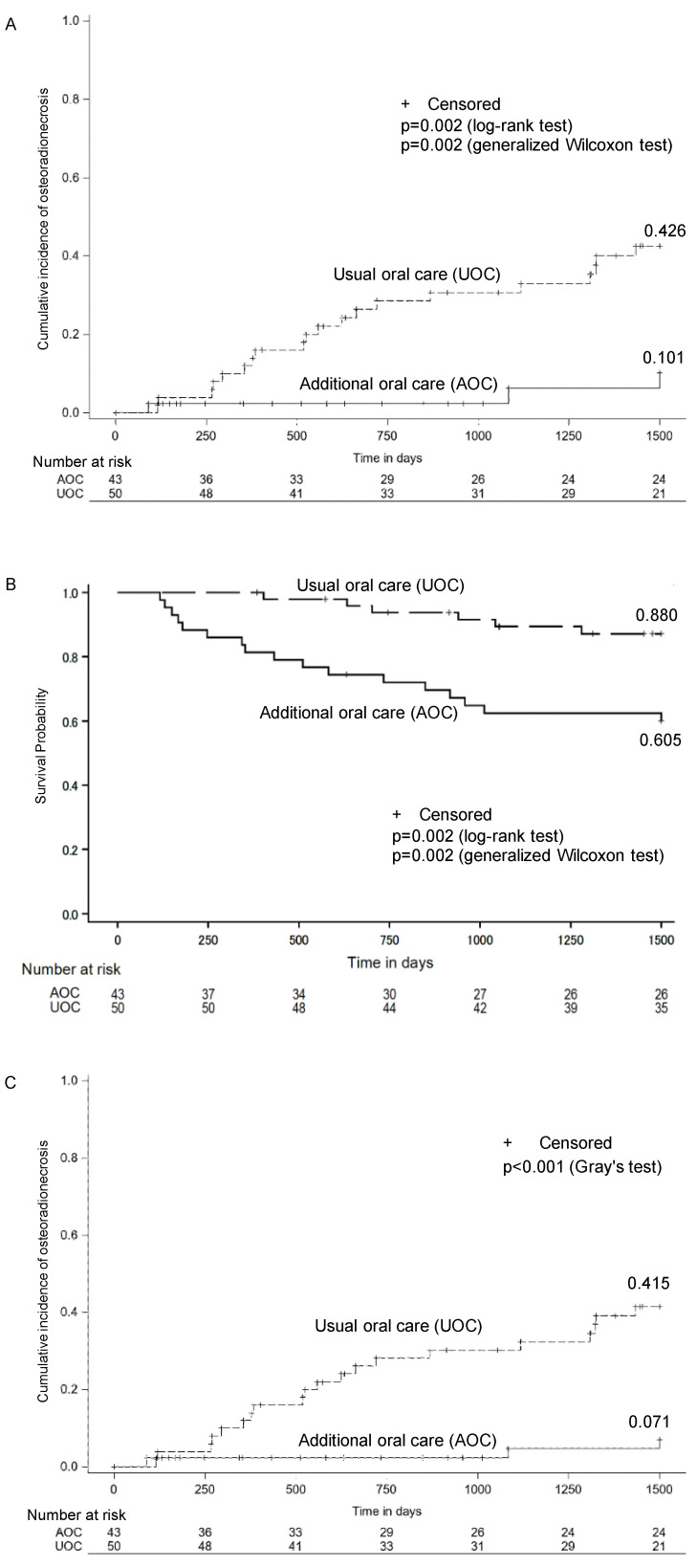
Cumulative incidence of osteoradionecrosis. A Results when death is not treated as competing risk of osteoradionecrosis. B Survival Probability by Treatment Group. C Results when death is treated as competing risk of osteoradionecrosis.

Figure 2 illustrates the overall survival and cumulative incidence of ORN for the AOC and UOC groups. Figure 2A displays the cumulative incidence of ORN estimated using the Kaplan-Meier method (without considering death as a competing risk). The incidence was lower in the AOC group (0.101) compared to the UOC group (0.426) (log-rank test, p = 0.002; generalized Wilcoxon test, p = 0.002). Figure 2B shows the Kaplan-Meier overall survival curves. The survival rate was lower in the AOC group compared to the UOC group (log-rank test, p=0.002; Wilcoxon test, p=0.002). Given the difference in survival, Figure 2C presents the cumulative incidence of ORN accounting for death as a competing risk using Gray's test. The incidence remained lower in the AOC group (0.071) compared to the UOC group (0.415) (p < 0.001).

Table 2 shows the results of the multivariate analysis. The HRs for the incidence of ORN in the AOC group versus the UOC group were lower in all three models: 0.122 (0.034-0.441, p = 0.001) in model 1, 0.110 (0.026-0.456, p = 0.002) in model 2, and 0.108 (0.019-0.606, p = 0.011) in model 3.

**Figure 3:**
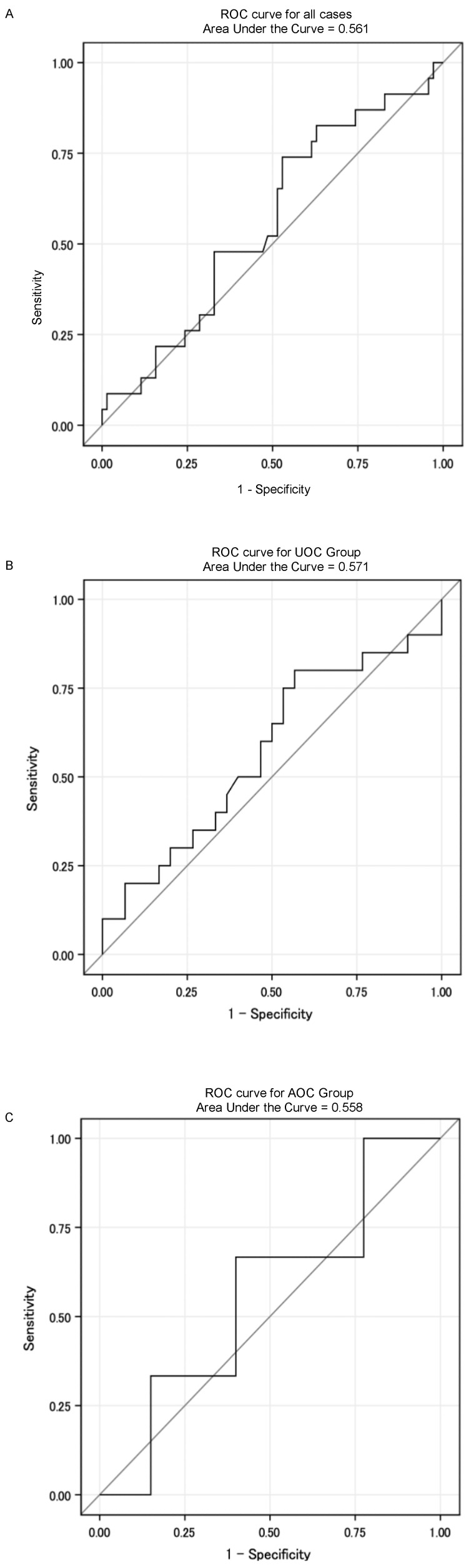
Receiver Operating Characteristic (ROC) curves for predicting osteoradionecrosis (ORN) using mean mandibular dose. A ROC curve for all patients (AUC = 0.561). B ROC curve for the Usual Oral Care (UOC) group (AUC = 0.571). C ROC curve for the Additional Oral Care (AOC) group (AUC = 0.558).

Figure 3 presents the ROC curves assessing the ability of mean mandibular dose to predict ORN development. Figure 3A shows the ROC curve for all patients (AUC = 0.561). Figure 3B shows the ROC curve for the UOC group (AUC = 0.571), and Figure 3C shows the ROC curve for the AOC group (AUC = 0.558). The logistic regression analysis indicated that mean mandibular dose was not a statistically significant predictor of ORN development (p = 0.3103).

**Figure 4:**
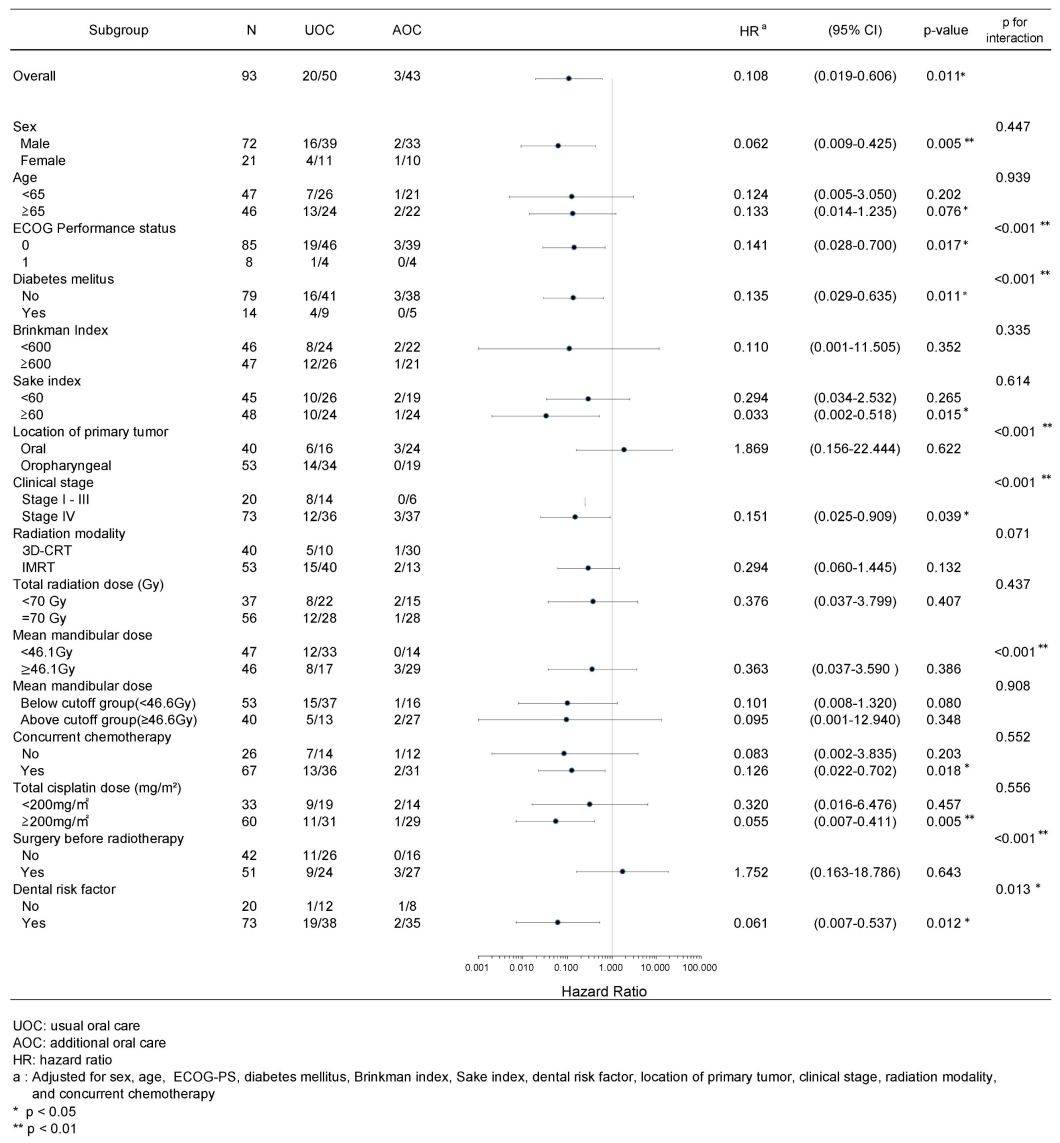
Subgroup analysis for osteoradionecrosis.

Figure 4 shows the results of the subgroup analysis. The HRs were lower for the following eight factors: 0.062 (0.009-0.425) for males, 0.141 (0.028-0.700) for ECOG-PS (PS-0), 0.135 (0.029-0.635) for diabetes mellitus (no), 0.033 (0.002-0.518) for the Sake index (≥60), 0.151 (0.025-0.909) for clinical stage (IV), 0.126 (0.022-0.702) for concurrent chemotherapy, 0.055 (0.007-0.411) for total cisplatin dose (≥200 mg/m^2^), and 0.061 (0.007-0.537) for DRF. The interaction was statistically significant for ECOG-PS (p < 0.001), diabetes mellitus (p < 0.001), location of the primary tumor (p < 0.001), clinical stage (p < 0.001), surgery before radiotherapy (p < 0.001), and DRF (p = 0.013).

## Discussion

In this study, the cumulative incidence of ORN was lower in the AOC group than in the UOC group, and similarly lower when death was analyzed as a competitive risk of developing ORN (Figure 2A, 2C). Liao et al. reported an association between tooth extraction and ORN development before and after radiation therapy 26, but this is the first study to report the outcome of treating death as a competitive risk of developing ORN with and without it. In this study, there was a difference in mortality between the two groups during the observation period (p=0.003). In addition, there was a difference in survival between the two groups (p=0.002 for the log-rank test and p=0.002 for the Wilcoxon test) (Figure 2B).

Therefore, we used death as a competitive risk to analyze the cumulative incidence of ORN. In this study, when death was not analyzed as a competing risk, the cumulative incidence rates were 42.6% in the UOC group and 10.1% in the AOC group. However, when death was analyzed as a competing risk, the cumulative incidence rate decreased to 41.5% and 7.1%, respectively, and the difference between the two groups increased by 1.9%, from 32.5% to 34.4%. These results suggest that the difference in cumulative incidence rates may be biased when death is not analyzed as a competing risk.

In this study, a multivariate analysis was conducted with smoking and alcohol consumption as risk factors were analyzed as covariates. To our knowledge, this is the first study that death was analyzed as a competing risk for ORN development and smoking and alcohol consumption were analyzed as covariates. Three models were used in the multivariate analysis. The HRs were in all models and had nearly the same values: HR of 0.122 in model 1 adjusted for patients' factors, 0.110 in model 2 adjusted further for cancer factors, and 0.108 in model 3 adjusted further for treatment factors (Table 2). The extraction of all at-risk teeth 2 weeks before radiotherapy reduced the risk of ORN development by approximately 90%. Previous reports on HR for ORN development due to tooth extraction before radiotherapy have been mixed, with some reports exceeding 1.0 and others below 1.0. Reports of HRs <1.0 were limited, such as 0.62 (0.27-1.47) by Kojima et al. In the present study, the HR study was very low. As noted earlier, the treatment of death as a competing risk in this study may have resulted in smaller HRs than in other studies. Another explanation for the very low HR could be bias due to factors that were not adjusted for between the two groups. In our study, we adjusted for a variety of factors, including patient factors, cancer factors, and treatment factors. Although there was no difference in the number of dentists or frequency of practice in our study, we cannot rule out the possibility that factors affecting the development of ORN were not adjusted for.

Marx described that radiotherapy-exposed mandibles become a tissue of with osteocytes and hypovascular and hypoxic, with increased infection risk and impaired wound healing, resulting in ORN development as a chronic intractable trauma 5. Delanian et al. described the pathophysiology of radiation-induced fibrosis and atrophy in ORN 39. They explained that radiotherapy-induced fibrosis is a process of radiation-induced vascular destruction, followed by an increase in fibroblasts, which eventually leads to a hypocellular bone tissue that leaves only weak fibrous tissue, making it susceptible to damage from trauma and infection. On the basis of the above explanations and the results of the present study, tooth extraction before radiotherapy may have reduced the risk of ORN development by removing factors that prevent healing from radiation-induced fibrosis.

The association between tooth extraction before and after radiotherapy and the risk of ORN development have been reported in several systematic reviews 33,40-45; however, the diversity of studies in terms of the duration of observation, different methods of radiotherapy, and criteria and timing of tooth extraction make comparisons difficult. In addition, in observational studies, what is adjusted for as a covariate has a effect on the results. In the future, when examining the risk of ORN development because of tooth extractions, death must be considered a competing risk for ORN development and risk factors appropriately as covariates, and these should be described so that they can be confirmed.

Subgroup analysis showed lower HRs of 1.0 for male sex, ECOG-PS (PS-0), absence of diabetes mellitus, alcohol consumption, clinical stage, concurrent chemotherapy, total cisplatin dose, and DRF (Figure 4). In these groups, removal of at-risk teeth before radiotherapy is expected to reduce the risk of ORN development. On the contrary, the following factors had HRs of <1.0; however, their HRs differed within subgroups: alcohol consumption (0.294 vs. 0.033), concurrent chemotherapy (0.083 vs. 0.126), and total cisplatin dose (0.320 vs. 0.055). Although all of these results were not significant, the HRs tended to be lower in the subgroups with more alcohol consumption and higher total cisplatin doses. These results appear to be contradictory because these factors were considered risk factors for ORN development in previous reports. Alcohol consumption has been shown to have a negative impact on oral hygiene 48. Therefore, patients with a high Sake index may have had poor oral hygiene. The addition of AOC to usual oral care may have improved oral hygiene and reduced the risk of ORN development, resulting in a large difference in HRs. These subgroups may have included more patients with good performance status, i.e., tolerate side effects of chemotherapy, which may have reduced the HRs. Concurrent chemoradiotherapy is a factor that worsens the oral hygiene condition by increasing adverse events such as oral mucositis 49. Therefore, the addition of AOC to usual oral care before radiotherapy may have improved the oral hygiene status and lowered the HR in the intervention group (AOC).

Similarly, cisplatin has a radiosensitizing effect, with 100 mg/m2 of cisplatin reported to be equivalent to 7.2 Gy 50. Therefore, high-dose cisplatin may increase the effect of radiotherapy on the oral cavity and worsen the oral hygiene condition. The addition of AOC to usual oral care prior to radiotherapy may have improved the oral hygiene condition and further reduced the HR in the intervention group (AOC).

Subgroup analysis showed lower HRs of 1.0 for male sex, ECOG-PS (PS-0), absence of diabetes mellitus, alcohol consumption, clinical stage, concurrent chemotherapy, total cisplatin dose, and DRF (Figure 4). In the subgroup with dental risk factors, the risk was eliminated in the AOC group based on the definition of Schiødt M et al. which may have further reduced the risk of developing ORN. Indeed, recent studies focusing on tooth-level factors have identified specific predictors for adverse dental outcomes after radiotherapy. Lalla RV et al. 51, for example, found that factors such as pre-radiotherapy bone loss, caries, and high-dose radiation to the tooth increased the risk of tooth loss or exposed bone, underscoring the importance of detailed pre-treatment dental assessment and risk stratification in identifying teeth for prophylactic extraction, as was performed in our AOC group based on the criteria.

The following subgroups had HRs well above 1.0: namely, 1.869 for the location of the primary tumor (oral) and 1.752 for surgery before radiotherapy. Wang et al. reported that oral cancers have a higher incidence of ORN than oropharyngeal or hypopharyngeal cancers 6. According to Monnier et al., surgery of the mandible before radiation therapy is a risk factor for ORN development 7.

Furthermore, we investigated the predictive value of mean mandibular dose for ORN using ROC analysis (Figure 3). The AUC was low (0.561 for all patients), and mean mandibular dose was not a significant predictor in logistic regression (p=0.3103). This suggests that while mean mandibular dose might contribute to ORN risk as reported by Aarup-Kristensen et al. 52, its predictive power might be limited in our cohort, possibly due to the strong influence of other factors or the specific characteristics of our patient population. Setting a clear cutoff value based solely on mean dose appears challenging. Future studies should explore multivariate models incorporating mean dose with other factors.

Considering the above reports, the HR was thought to be higher when the primary tumor was located in the oral cavity than when present in the oropharynx because of the direct effect of the cancer on the mandible and the higher irradiation dose to the mandible. Performing surgery before radiotherapy was also considered to have a higher HR because the direct invasion of the mandible by surgery delays the recovery from the damage caused by radiotherapy.

This study has five strengths. First, the analysis was performed with death as a competing risk for ORN development. Second, multivariate analysis was performed with death as a competing risk and smoking and alcohol consumption as covariates. Third, a subgroup analysis was performed to identify groups that would benefit from tooth extraction before radiotherapy. Fourth, the rate of prognostic information was high. The median observation period for both groups was 1,500 days, which means that more than half of the patients were followed up until the end of the observation period. Fifth, this study clearly defined the criteria for tooth extractions and when to perform them. Few previous reports have clearly stated the criteria for tooth extraction.

However, this study has five limitations. First, this was a single-center study, making it difficult to generalize the results. Second, because of the small sample size, many factors were not statistically significant in the subgroup analysis. Third, this was an observational study and may have been influenced by factors that were not adjusted between the two groups. Fourth, the incidence of ORN after 1,500 days could not be determined because of the study's observation period of 1,500 days. Finally, limited detailed dental data is a limitation. AOC group data from paper records before 2015 made retrospective verification difficult. Consequently, precise data on extraction number, timing, and tooth location were unavailable, hindering nuanced analysis of their impact on ORN risk. Future studies should prioritize prospective, detailed dental data collection. It is also important to consider the potential impact of the number of teeth extracted, although detailed data was unavailable in our study. Recent research has highlighted potential factors influencing ORN risk after pre-radiotherapy extractions. While detailed data on the number of extractions was unavailable in our study, Tsu-Jen Kuo et al. 53 reported that extracting a higher number of teeth might be associated with an increased risk of ORN. Furthermore, inflammatory markers before treatment, as investigated by Yilmaz et al. 54, could also play a role in subsequent ORN development. These findings underscore the complexity of balancing the benefits of removing infectious foci with the potential risks associated with extensive dental procedures and prolonged inflammation before radiotherapy. This highlights the need for future studies to carefully evaluate the optimal number and timing of extractions, potentially stratifying patients based on both dental and systemic inflammatory status, to balance risk reduction and potential harm.

## Conclusion

This observational study of patients with locally advanced oral and oropharyngeal cancer who received radiotherapy alone or chemoradiotherapy revealed that the group that underwent extraction of at-risk teeth 2 weeks before radiotherapy had a lower incidence of ORN than the group that did not undergo extraction. This result remained unchanged when death was analyzed as a competing risk for ORN development. Results of a multivariate analysis including patient factors such as smoking and alcohol consumption, cancer factors, and treatment factors showed that the extraction of at-risk teeth before radiotherapy reduced the risk of ORN development by approximately 90%. However, these results are based on a retrospective observational study conducted at only one center. Thus, future multivariate studies conducted at multiple centers, which analyzed death as a competing risk, are warranted.

## Figures and Tables

**Table 1:**
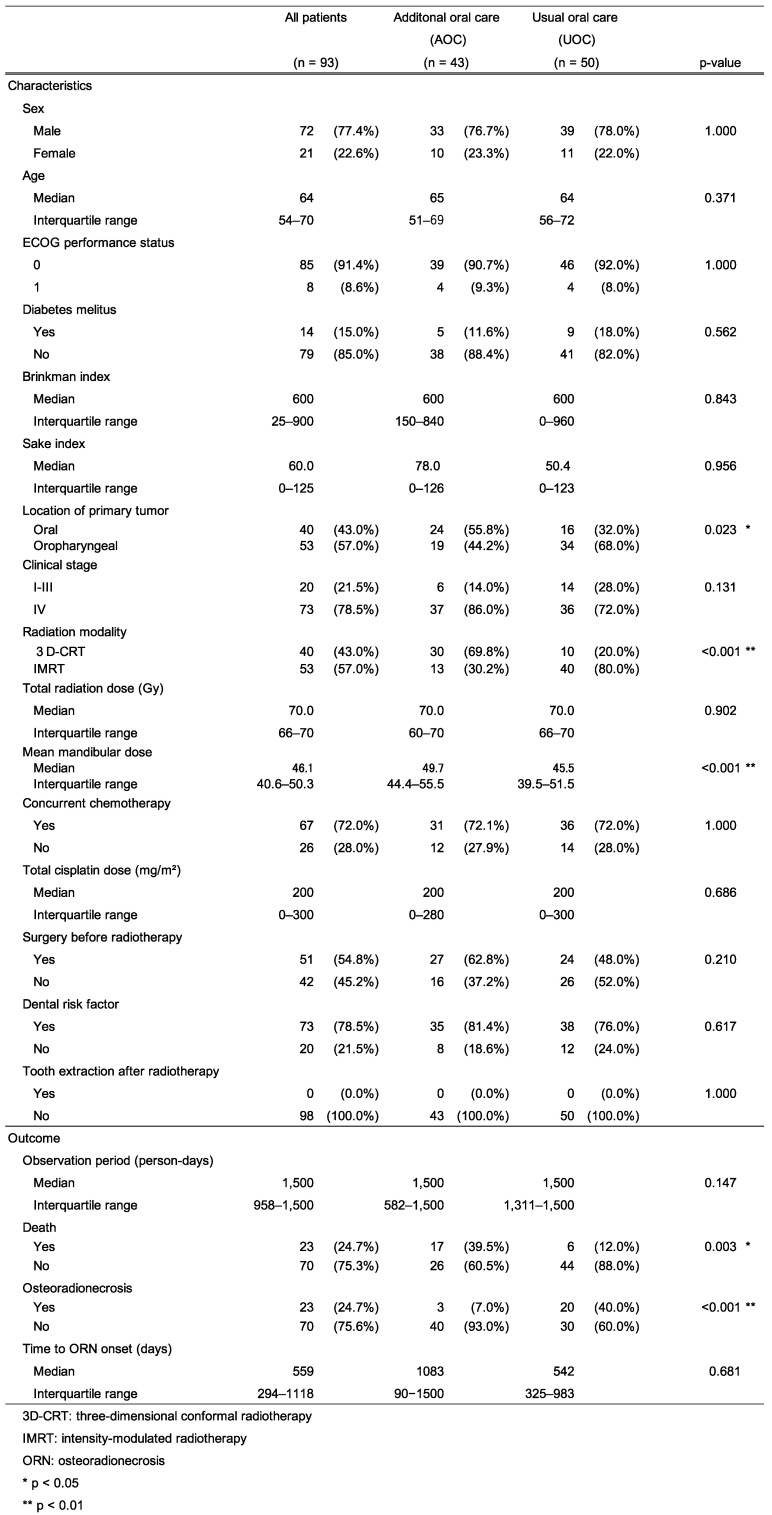
Characteristics and outcome of patients in the analysis

**Table 2:**
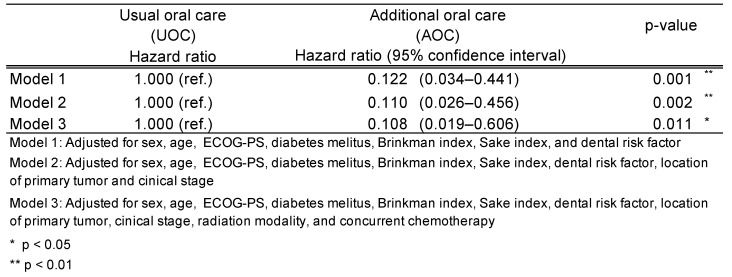
Results of multivariate regression analysis for osteoradionecrosis

## Abbreviations

3w-CDDP + RT: chemoradiotherapy with 3-weekly cisplatin
ORN: osteoradionecrosis
RCT: randomized controlled trial
NCCN: National Comprehensive Cancer Network
3D-CRT: three-dimensional conformal radiotherapy
HR: hazard ratio
CI: confidence interval
MCC: Miyagi Cancer Center
DRF: dental risk factor
MRRF: malignancy-related risk factor
AOC: additional oral care
UOC: usual oral care
IMRT: intensity-modulated radiation therapy
ECOG-PS: Eastern Cooperative Oncology Group performance status
